# Submission for Special Issue: The Role of Platelet Activation in the Pathophysiology of HIV, Tuberculosis, and Pneumococcal Disease. Bedaquiline Suppresses ADP-Mediated Activation of Human Platelets *In Vitro via* Interference With Phosphatidylinositol 3-Kinase

**DOI:** 10.3389/fimmu.2020.621148

**Published:** 2021-02-26

**Authors:** Gregory R. Tintinger, Annette J. Theron, Helen C. Steel, Moloko C. Cholo, Jan G. Nel, Charles Feldman, Ronald Anderson

**Affiliations:** ^1^ Department of Internal Medicine, Steve Biko Academic Hospital and Faculty of Health Sciences, University of Pretoria, Pretoria, South Africa; ^2^ Department of Immunology, Faculty of Health Sciences, University of Pretoria, Pretoria, South Africa; ^3^ Department of Haematology, Faculty of Health Sciences, University of Pretoria, Pretoria, South Africa; ^4^ Tshwane Academic Division, National Health Laboratory Service of South Africa, Pretoria, South Africa; ^5^ Department of Internal Medicine, Faculty of Health Sciences, University of the Witwatersrand, Johannesburg, South Africa

**Keywords:** adenosine-5′-triphosphate, bedaquiline, calcium fluxes, CD62P, phosphatidylinositol 3-kinase, platelets, wortmannin

## Abstract

Although bedaquiline has advanced the treatment of multidrug-resistant tuberculosis (TB), concerns remain about the cardiotoxic potential of this agent, albeit by unexplored mechanisms. Accordingly, we have investigated augmentation of the reactivity of human platelets *in vitro* as a potential mechanism of bedaquiline-mediated cardiotoxicity. Platelet-rich plasma (PRP) or isolated cells prepared from the blood of healthy, adult humans were treated with bedaquiline (0.625–10 µg/ml), followed by activation with adenosine 5’-diphosphate (ADP), thrombin or the thromboxane A_2_ receptor agonist (U46619). Expression of platelet CD62P (P-selectin), platelet aggregation, Ca^2+^ fluxes and phosphorylation of Akt1 were measured using flow cytometry, spectrophotometry, fluorescence spectrometry, and by ELISA procedures, respectively. Exposure to bedaquiline caused dose-related inhibition of ADP-activated, but not thrombin- or U46619-activated, expression of CD62P by platelets, achieving statistical significance at a threshold concentration of 5 µg/ml and was paralleled by inhibition of aggregation and Ca^2+^ mobilization. These ADP-selective inhibitory effects of bedaquiline on platelet activation were mimicked by wortmannin, an inhibitor of phosphatidylinositol 3-kinase (PI3-K), implicating PI3-K as being a common target of both agents, a contention that was confirmed by the observed inhibitory effects of bedaquiline on the phosphorylation of Akt1 following activation of platelets with ADP. These apparent inhibitory effects of bedaquiline on the activity of PI3-K may result from the secondary cationic amphiphilic properties of this agent. If operative *in vivo*, these anti-platelet effects of bedaquiline may contribute to ameliorating the risk of TB-associated cardiovascular disease, but this remains to be explored in the clinical setting.

## Introduction

Inclusion of the diarylquinoline agent, bedaquiline, in chemotherapeutic regimens used in the treatment of multidrug-resistant and extensively drug-resistant (MDR/XDR)-tuberculosis (TB) has resulted in significantly improved outcomes (1–5). The therapeutic efficacy of bedaquiline has been attributed to its novel mechanism of action, targeting the F_1_/F_0_ ATP synthase of the *Mycobacterium tuberculosis (Mtb)* respiratory chain, encompassing both replicating and non-replicating bacilli (1, 2). Concerns remain, however, about the risk for development of cardiac dysfunction during chemotherapy with bedaquiline, specifically prolongation of the cardiac QT interval, an indicator of development of ventricular tachyarrhythmias and possibly cardiac arrest (2, 6–10). The risk of QT interval prolongation may be cumulative when bedaquiline is used in combination with other components of MDR/XDR-TB regimens, specifically clofazimine and delamanid (9, 10).

Although largely unexplored, acquisition of insights into the pathogenesis of bedaquiline-associated adverse cardiac events may enable the development of strategies to ameliorate risk. This may be of considerable importance since TB *per se* is also associated with a chronic pro-inflammatory/pro-thrombotic state and increased risk of acute cardiovascular events (11) that may be exacerbated in the settings of extended chemotherapy and co-existent co-morbidities (12). To date, however, only very limited data exists with respect to identification of mechanisms that underpin the cardiotoxic potential of bedaquiline. In this context, one study has described the potent inhibitory effects of bedaquiline on the activity of the Kv.11.1 channel (4), which mediates the repolarizing current in the cardiac action potential. However, the clinical relevance of this study, which was undertaken using Kv.11.1 channel-transfected hamster embryonic kidney cells *in vitro*, remains to be established (4).

An alternative, apparently unexplored, mechanism of bedaquiline-associated cardiac dysfunction relates to possible augmentative effects of this agent on the reactivity of human platelets. These cells not only drive inflammation and thrombosis (13, 14), but have also been implicated in the pathogenesis of cardiac arrhythmias (15). Accordingly, investigation of the effects of bedaquiline on the responses of human platelets activated *in vitro* with adenosine 5’-diphosphate (ADP), thrombin or a thromboxane A_2_ receptor agonist, represent the primary focus of the current study. As a strategy to identify possible mechanisms of action, the effects of bedaquiline on platelet responses activated by the various receptor-mediated stimuli were compared with those of two pharmacological antagonists of intracellular signaling pathways, specifically U73122 and wortmannin, inhibitors of phospholipase C (PLC) and phosphatidylinositol 3-kinase (PI3-K), respectively. In addition, we also investigated the effects of bedaquiline on PI3-K-mediated phosphorylation of the serine/threonine kinase, Akt1, the predominant isoform in human platelets (16) and key mediator of platelet α-granule release (17).

## Materials and Methods

### Ethics Committee Approval

Permission to undertake this study and draw blood from healthy, adult human volunteers was granted by the Research Ethics Committee of the Faculty of Health Sciences, University of Pretoria in full compliance with the World Medical Association Declaration of Helsinki, 2013 (Approval number: 116/2017). Informed consent was obtained from all participants in the study prior to the blood draw [17 female:7 male, mean age ( ± SD) = 35.25 ± 12.0 years].

### Test Agents

Bedaquiline, (1*R*,2*S*)-1-(6-bromo-2-methoxyquinolin-3-yl)-4-(dimethylamino)-2-naphthalen-1-yl-1-phenylbutan-2-ol, was purchased from AdooQ Bioscience (York, UK); wortmannin [(*1R,3R,5S,9R*,18S)-18-(methoxymethyl)-1,5-dimethyl-6,11,16-trioxo-13,17-dioxapentacyclo[10.6.1.0^2,10^.0^5,9^.0^15,19^]nonadeca-2(10),12(19),14-trien-3-yl] acetate, a selective inhibitor of PI3-K, from the Sigma Chemical Co. (St Louis. MO, USA); U73122, {1-6[[(17ß)-3-methoxyestra-1,3,5(10)-trien-17-yl]amino]hexyl]-1*H*-pyrolle-2,5-dione}, a selective inhibitor of phospholipase C (PLC), and U46619, (*Z*)-7-{[1*R*,4*S*,5*S*,6*R*]-6-[(*E*,3*S*)-3-hydroxyoct-1-enyl]-2-oxabicyclo[2.2.1]heptan-5-yl}hept-5-enoic acid, a thromboxane A_2_ surrogate, were purchased from Tocris Bioscience, Bristol, UK. Bedaquiline, wortmannin, U73122, and U46619 were dissolved to stock concentrations in dimethylsulfoxide (DMSO), and used at final concentrations of 0.625–10 µg/ml (equivalent to 1.13–18 µM), 1 µM and 0.625 µM and 5 µM, respectively, in the assays of platelet activation described below, which also included appropriate solvent controls.

Adenosine-5’-diphosphate (ADP) and thrombin (from human plasma) were purchased from Boehringer Mannheim Biochemical (Basel, Switzerland) and the Sigma Chemical Co. respectively and used at final maximal concentrations of 100 µM and 1 NIH unit/ml respectively. ADP is an agonist of platelet P2Y1 and P2Y12 receptors and acts as a secondary, autocrine activator of these cells, while thrombin is an agonist of the platelet proteinase-activated receptors 1 and 4 (PAR1/4). U46619 was used at a final concentration of 5 µM, as mentioned above.

Unless indicated, all other chemicals and reagents were purchased from the Sigma Chemical Co.

### Platelet-Rich Plasma

To prepare platelet-rich plasma (PRP), blood (anti-coagulated with five units of preservative-free heparin/ml blood) was centrifuged at 250 x g for 10 min at room temperature within 15 min of venepuncture and the essentially erythrocyte- and leukocyte-free upper layer of PRP decanted and used in the experiments described below.

### Purified Platelet Suspensions

PRP was diluted four-fold with phosphate-buffered saline (PBS, 0.15M, pH 6.8) containing ethylene glycol-bis(β-aminoethyl ether)-N-N-N’-N’-tetraacetic acid (EGTA, final concentration 0.6 mM) and centrifuged at 260 x g for 10 min at 20°C to deplete residual erythrocytes and leukocytes. The platelet-enriched supernates were then centrifuged at 800 x g for 20 min and the cell pellets resuspended in PBS (pH 7.4) and counted using a Sysmex XN 20000 Automated Haematology Analyzer (Sysmex Corporation, Kobe, Japan).

The low pH (6.8) of the PBS together with the addition of EGTA to deplete extracellular Ca^2+^ and preparation of the cells at a constant temperature of 20°C, were used to minimize platelet activation. We preferred this strategy to inclusion of acetylsalicyclic acid as we have previously observed that induction of synthesis of thromboxane A_2_ represents a significant component of the platelet response following activation with ADP.

### Effects of Bedaquiline on Platelet Activation According to the Magnitude of Expression of CD62P

Platelet activation was measured by flow cytometry as the proportion (%) of CD42a^+^ platelets expressing the α-granule-derived adhesion molecule, CD62P (P-selectin), a key mediator of platelet activation and pro-thrombotic activity. For these experiments, PRP (20 µl) was added to 980 µl of Hanks’ balanced salt solution (HBSS, indicator-free, 1.25mM calcium, pH7.4) and incubated for 10 min at 37°C in the presence of bedaquiline (0.625–10 µg/ml final, equivalent to 1.13–18 µM), or wortmannin (1µM), or U73122 (0.625 µM), or an equal volume of DMSO to control systems (0.1–0.2%, final). This was followed by the addition of ADP, thrombin, U46619 or an equal volume of HBSS (background to detect spontaneous platelet activation). The tubes were then incubated for a further period of 5 min at 37°C and processed immediately thereafter for analysis by flow cytometry. The cells were stained with 5 µl each of a murine anti-human platelet CD42a-phycoerythrin (PE)-labeled monoclonal antibody (Becton Dickenson, Franklin Lakes, USA) and an anti-human CD62P-fluorescein isothiocyanate (FITC)-labeled monoclonal antibody (Beckman Coulter, Miami, FL, USA) to detect the total and activated platelet populations respectively. After 15 min of incubation in the dark, the samples were analyzed on a Gallios flow cytometer (Beckman Coulter, Miami, FL, USA) and the results expressed as the percentage of activated platelets with 50,000 cells interrogated during each measurement.

### Measurement of Platelet Cytosolic Ca^2+^ Fluxes

Fura-2 acetoxymethyl ester (fura-2AM) was used as the fluorescent, Ca^2+^-sensitive indicator for these experiments (18). In this series of experiments, purified platelets suspended in HBSS were incubated at 37°C for 5 min followed by addition of fura-2AM (2 µM) and a further incubation period of 25 min at 37°C after which the platelets were pelleted by centrifugation at 350 x g for 20 min. The cell pellets were resuspended in PBS. For measurement of cytosolic Ca^2+^ fluxes, 100 µl of platelet suspension was added to 2.9 ml HBSS and incubated for 10 min at 37°C in the absence or presence of either bedaquiline (5 and 10 µg/ml) or wortmannin (1 µM). The fura-2AM-loaded platelets were then transferred to disposable reaction cuvettes maintained at 37°C in a Hitachi 650 10S fluorescence spectrometer with excitation wavelengths set at 340 and 500 nm respectively. After a stable baseline was obtained, the platelets were activated by addition of either ADP (100 µM) or thrombin (1 NIH unit/ml) and alterations in fluorescence intensity measured over a 5 min time course. These results are presented as the peak Ca^2+^ concentrations calculated as described previously (18) and as typical traces from representative experiments.

### Measurement of Platelet Aggregation

For this limited series of experiments, PRP (150 µl), or an equal volume of platelet poor plasma (PPP) prepared by centrifugation of PRP at 350 x g for 5 min, was added to 1,850 µl of HBSS followed by the addition of 2 µl of bedaquiline (10 µg/ml final), wortmannin (1µM final), or DMSO to control systems and the test tubes incubated at 37°C for 20 min. Thereafter, ADP (100 µM final) or an equal volume of HBSS (background to detect spontaneous platelet aggregation), were added to the tubes, which were incubated at 37°C for 10 min with repeated inversion at 1-min intervals. For measurement of platelet aggregation, 250 µl from each tube (3 repeats) were added to a 96-well microtissue culture plate using reverse pipetting to minimize air bubbles. Light absorbance, as a surrogate of platelet aggregation, was measured spectrophotometrically at a wavelength of 650nm (19). The results are expressed as the mean percentage aggregation, which was calculated based on the absorbance of the PPP control (100% aggregation) and the PRP control (0% absorbance).

### Measurement of Akt1 Phosphorylation

Platelets (approximately 1 x 10^8^/ml) suspended in HBSS were preincubated with bedaquiline (5 and 10 µg/ml) or an equivalent volume of DMSO for 10 min at 37°C followed by the addition of ADP (100 µM). After 3 min of incubation (20–22), the reactions were terminated and the platelets lysed by addition of an equal volume of cell extraction buffer supplemented with a 1% protease and phosphatase inhibitor cocktail (all purchased from Invitrogen, Thermo Fisher Scientific, Carlsbad, CA, USA) and held on ice for 30 min. Thereafter, the cell extracts were centrifuged at 13,000 rpm for 10 min and the supernatants decanted and stored at −80°C until analysis of phosphorylated Akt1 using an Ultrasensitive solid phase sandwich ELISA system (Thermo Fisher Scientific). The results of five experiments using platelets from three separate donors are expressed as units/ml.

### Expression and Statistical Analysis of Results

The results of each series of experiments are expressed as the median values with interquartile ranges, with the exception of platelet counts, contaminating cells, platelet aggregation and cytosolic Ca^2+^ fluxes, which are expressed as the mean values ± standard deviations, for a minimum of 3–4 different donors with numbers of replicate experiments indicated. Statistical analysis was performed using WinStat statistical software with the Shapiro-Wilk test used to test the distribution of data and levels of statistical significance calculated using the Mann-Whitney U-test for comparison of non-parametric data. A *P* value of <0.05 was considered significant.

## Results

### Platelet Counts

Circulating platelet counts for all blood donors were within the normal range (150–400 x 10^9^/l), while the mean number of platelets in the concentrated, purified platelet suspensions was 558 x 10^9^/l (n=14). The numbers of contaminating neutrophils, lymphocytes and monocytes in the pure platelet suspensions were almost undetectable, the mean values with SDs being 0.98 ± 0.14. 0.23 ± 0.42 and 0.016 ± 0.02 x 10^9^, respectively.

### Effects of Bedaquiline, Wortmannin, and U-73122 on ADP-, Thrombin-, and U46619-Activated Expression of CD62P by Platelet-Rich Plasma

**Figure 1:**
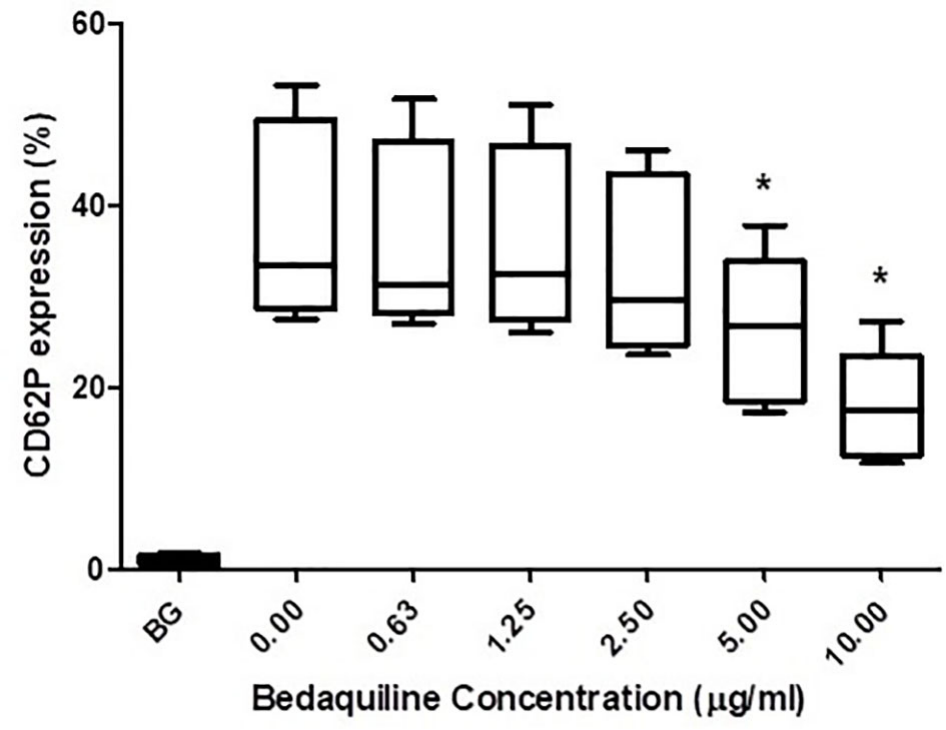
Effects of bedaquiline (BDQ, 0.625–10 µg/ml) on the magnitude of CD62P expression on platelets activated with adenosine diphosphate (ADP, 100 µM). The results are expressed as the median % CD62P expression with interquartile ranges (n=10 experiments). *P<0.05 for comparison of BDQ (5 and 10µg/ml) respectively, with the control system (BG = unstimulated platelets).

**Figure 2:**
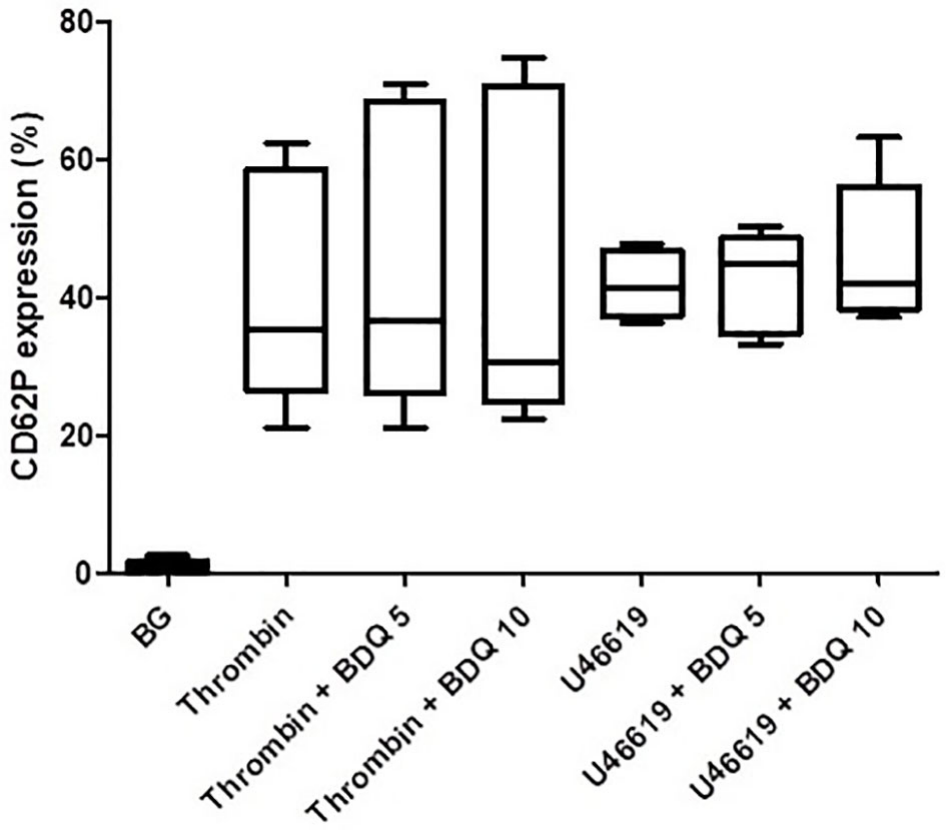
Effects of bedaquiline at 5 and 10 µg/ml on the magnitude of CD62P expression on platelets activated by thrombin (1 NIH unit/ml) or U46619 (5 µM). The results are expressed as the median % CD62P expression with interquartile ranges (data from nine and six experiments), respectively. No statistically differences were evident on comparison of the bedaquiline-untreated and –treated systems at both concentrations with both activators.

As shown in **Figure 1**, pre-treatment of PRP with bedaquiline (0.625–10 µg/ml) resulted in dose-related inhibition of ADP (100 µM)-activated upregulation of expression of CD62P, which achieved statistical significance at concentrations of 5 and 10 µg/ml bedaquiline (*P*<0.05). As shown in **Figure 2**, however, the corresponding responses of platelets activated with thrombin (1NIH unit/ml) or U46619 (5µM) were unaffected by exposure to bedaquiline at either 5 or 10 µg/ml, demonstrating a selective inhibitory effect of the test anti-mycobacterial agent on ADP-mediated platelet activation.

**Figure 3:**
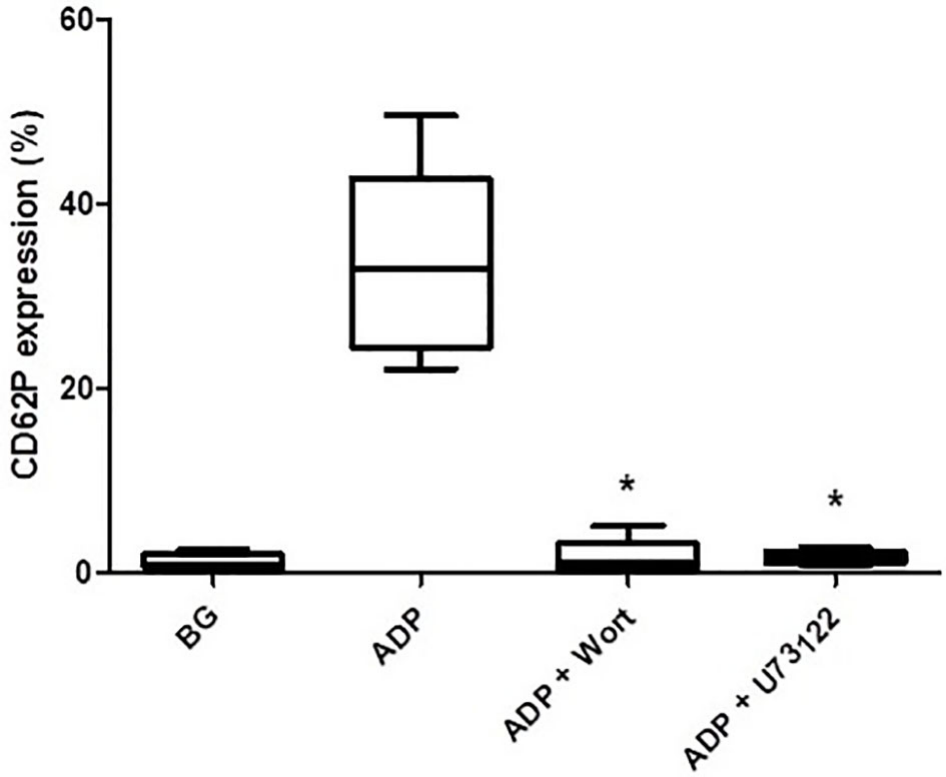
The effects of wortmannin (wort, 1 µM) and U73122 (0.625 µM) on the magnitude of CD62P expression on platelets activated with ADP (100 µM). The results are expressed as the median % CD62P expression with interquartile ranges (n=6–7 experiments). *P<0.05, for comparison of both the wortmannin- and U73122-treated systems with the corresponding control system. (BG = unstimulated platelets).

As shown in **Figure 3**, pre-treatment of PRP with either wortmannin (1 µM) or U73122 (0.625 µM), almost completely attenuated ADP-activated upregulation of CD62P expression, seemingly demonstrating the mechanistic involvement of PI3-K and/or PLC in this event.

**Figure 4:**
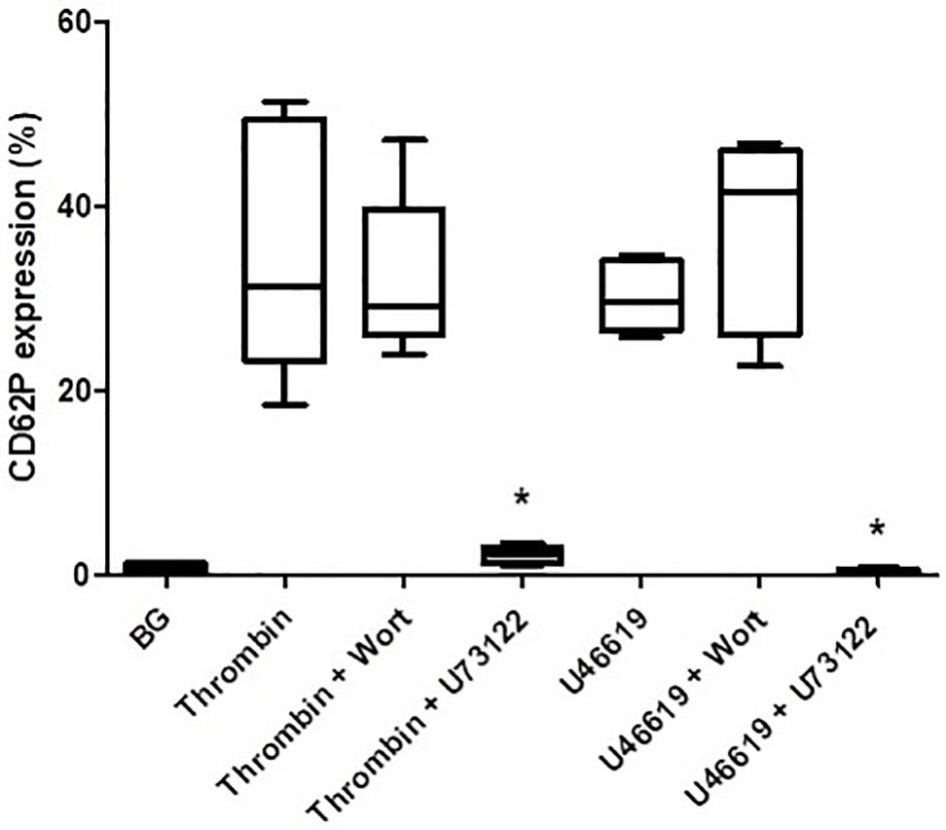
The effects of wortmannin (wort, 1 µM) or U73122 (0.625 µM) on the magnitude of CD62P expression on platelets activated with thrombin (1NIH unit/ml) or U46619 (5 µM). The results are expressed as the median % CD62P expression with interquartile ranges (n=6–12 experiments). *P<0.05, for comparison of U73122 with the corresponding control system. (BG = unstimulated platelets).

The effects of wortmannin (1 µM) or U73122 (0.625 µM) on thrombin- and U46619-activated platelets are shown in **Figure 4**. Pre-treatment of PRP with wortmannin did not affect the magnitude of either thrombin- or U46619-mediated upregulation of CD62P expression, while pre-treatment with U73122 almost completely attenuated upregulation of CD62P expression mediated by both thrombin and U46619. These results appear to demonstrate the key involvement of PLC, but not that of PI3-K, in upregulated expression of CD62P by platelets activated by thrombin and U46619.

### Effects of Bedaquiline on Platelet Cytosolic Ca^2+^ Fluxes Activated by ADP or Thrombin

**Figure 5:**
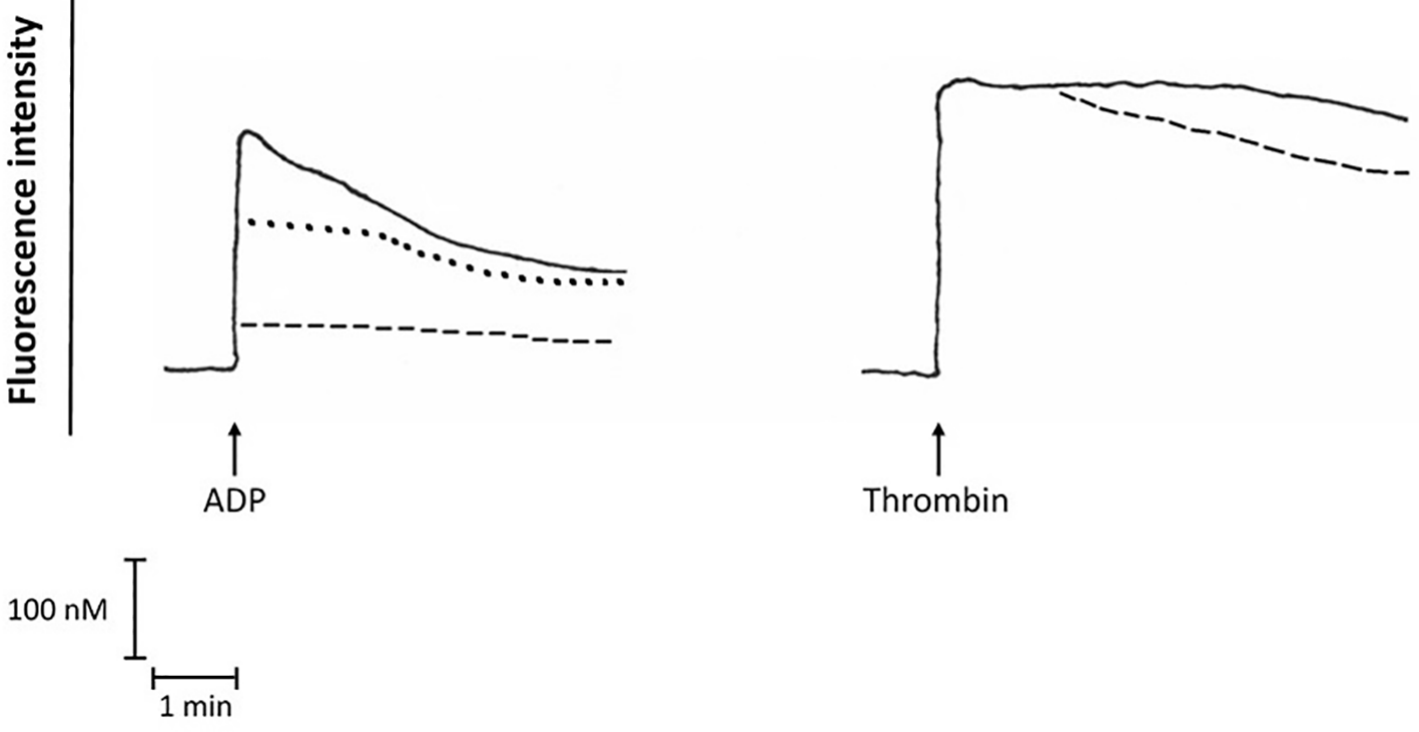
Fura-2 fluoresecence responses of ADP (100 µM)-activated platelets in the absence (**^:::^**) or presence of bedaquiline (BDQ, 10 µg/ml) (**- - - -**) or wortmannin (1 µM) (**^………^**), as well as the fura-2 fluorescence responses of thrombin (1 NIH unit/ml)-activated platelets in the absence (^:::^) or presence of BDQ (10 µg/ml) (**- - - -**). These are traces from a single representative experiment with a total of 5–12 in each series. The arrow (↑) denotes the addition of ADP or thrombin.

As shown in **Figure 5**, pre-treatment of purified platelets with bedaquiline at 10 µg/ml or wortmannin (1 µM) caused statistically significant inhibition of the immediate increase in the concentration of cytosolic Ca^2+^ following addition of ADP, while the more intense response activated by thrombin was unaffected by treatment of platelets with either of these agents. The basal cytosolic Ca^2+^ concentration of unstimulated platelets was 192 ± 21 nM, while the peak values observed for ADP-activated platelets in the absence or presence of bedaquiline or wortmannin were 420 ± 121 nM, 81 ± 74 nM (*P*<0.05) and 330 ± 136 nM (*P*<0.05), respectively. The corresponding values for thrombin-activated platelets in the absence or presence of bedaquiline were 680 ± 124 nM and 694 ± 151 nM (*P*=0.77), respectively (n=6–12 experiments). The slight increase in the rate of decline of fluorescence intensity in the thrombin-activated system in the presence of bedaquiline may result from attenuation of the endogenous ADP-mediated autocrine response.

These findings implicate interference with both PLC and PI3-K in bedaquiline-mediated inhibition of ADP-mediated activation of platelets.

### Effects of Bedaquiline and Wortmannin on Platelet Aggregation

Pre-treatment of platelets with bedaquiline (10 µg/ml) or wortmannin (1 µM) caused statistically significant inhibition of platelet aggregation. The magnitudes of ADP-activated platelet aggregation in the absence or presence of bedaquiline or wortmannin were 17, 3.7, and 0.4%, (*P*<0.05) (n=8–14 experiments), respectively. These findings reveal that bedaquiline inhibits several functions of ADP-activated platelets.

### Effects of Bedaquiline on ADP-Activated Phosphorylation of Akt1

**Figure 6:**
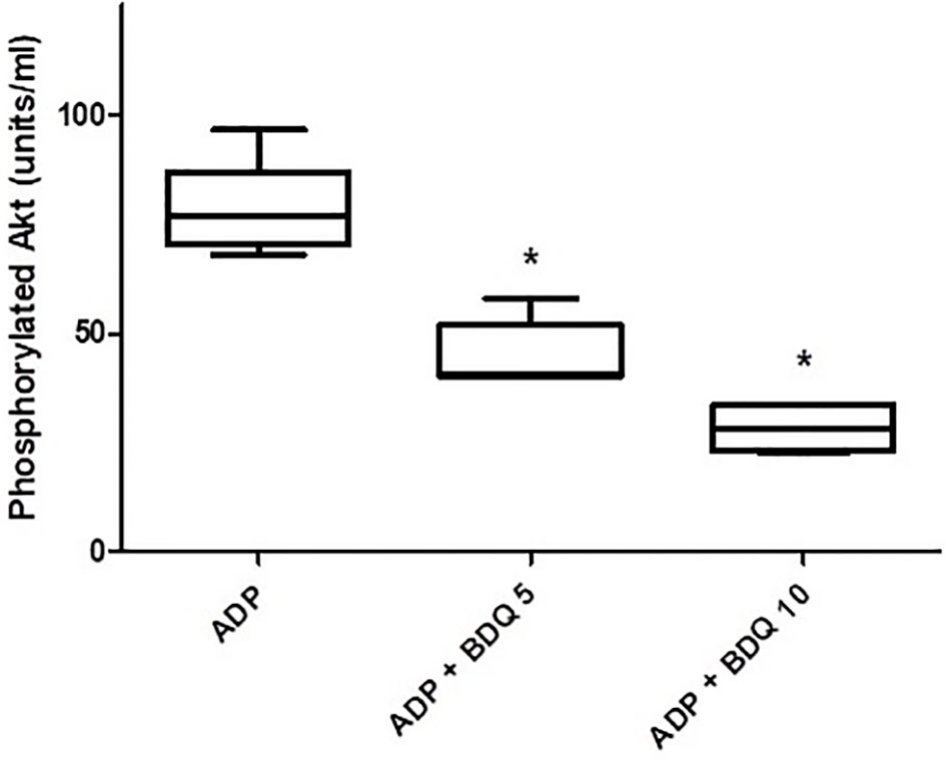
Effects of bedaquiline (5 and 10 µg/ml) on PI3-K-mediated phosphorylation of Akt1 measured in the lysates of ADP (100 µM)-activated platelets using an ELISA procedure. The results of five experiments (n=3 donors) are presented as the median values with interquartile ranges as units phosphorylated Akt1/ml; *P<0.05 for comparison of both bedaquiline-treated systems with the drug-free control system.

As shown in **Figure 6**, pre-treatment of isolated platelets with bedaquiline (5 and 10 µg/ml) resulted in potent, statistically significant (*P*<0.05 for both), dose-related inhibition of PI3-K-mediated phosphorylation of Akt1, representing the probable mechanism by which bedaquiline attenuates ADP-induced platelet activation.

## Discussion

Most importantly, this study has demonstrated that bedaquiline, at concentrations of 5–10 µg/ml, caused statistically significant inhibition of several activities of human platelets activated with ADP *in vitro*, but not with either thrombin or U46619 (a thromboxane A_2_ surrogate). These activities of platelets included upregulation of expression of CD62P, mobilization of intracellular Ca^2+^, and aggregation. Importantly, the concentrations of bedaquiline at which these inhibitory effects on platelet activation were observed are attainable during antimicrobial chemotherapy with this agent, reaching peak plasma concentrations of 5.5 µg/ml at 4–6 h following administration of the initial recommended dose of 400 mg/day (23).

From a mechanistic perspective, the absence of effects of bedaquiline on upregulation of CD62P expression by thrombin- or U46619-activated platelets excludes non-specific cytotoxicity as the cause of the selective inhibitory effects of the test agent on the responses of ADP-activated platelets. Moreover, inhibition of PLC and mobilization of intracellular Ca^2+^ as a putative primary mechanism of bedaquiline-mediated interference with ADP-activated platelet activation also seems improbable. This contention is based on the failure of bedaquiline to inhibit the responses of platelets activated with either thrombin or U46619. As with ADP, triggering of platelets by thrombin, thrombin receptor-activating peptides (TRAPs) and U46619 are dependent on activation of PLC and mobilization of intracellular Ca^2+^, as confirmed in the current study according to the stimulus-non-specific inhibitory effects of the potent PLC inhibitor, U73122.

The selective inhibitory effects of bedaquiline on ADP-activated platelets, included Ca^2+^ mobilization, CD62P expression and aggregation that were closely mimicked by the PI3-K inhibitor, wortmannin, suggesting that this enzyme *per se*, or related events downstream of its activation, may be the target of bedaquiline. In this context, PI3-K has been reported by others to be critically involved in ADP-mediated activation of platelets, including all of the aforementioned activities (24, 25), while the corresponding responses of platelets activated by thrombin, TRAPs, and U46619 are largely unaffected by inhibitors of PI3-K (26–28). This contention is supported by the observed potent inhibitory effects of bedaquiline on PI3-K-mediated phosphorylation of Akt1 following activation of platelets with ADP.

**Figure 7:**
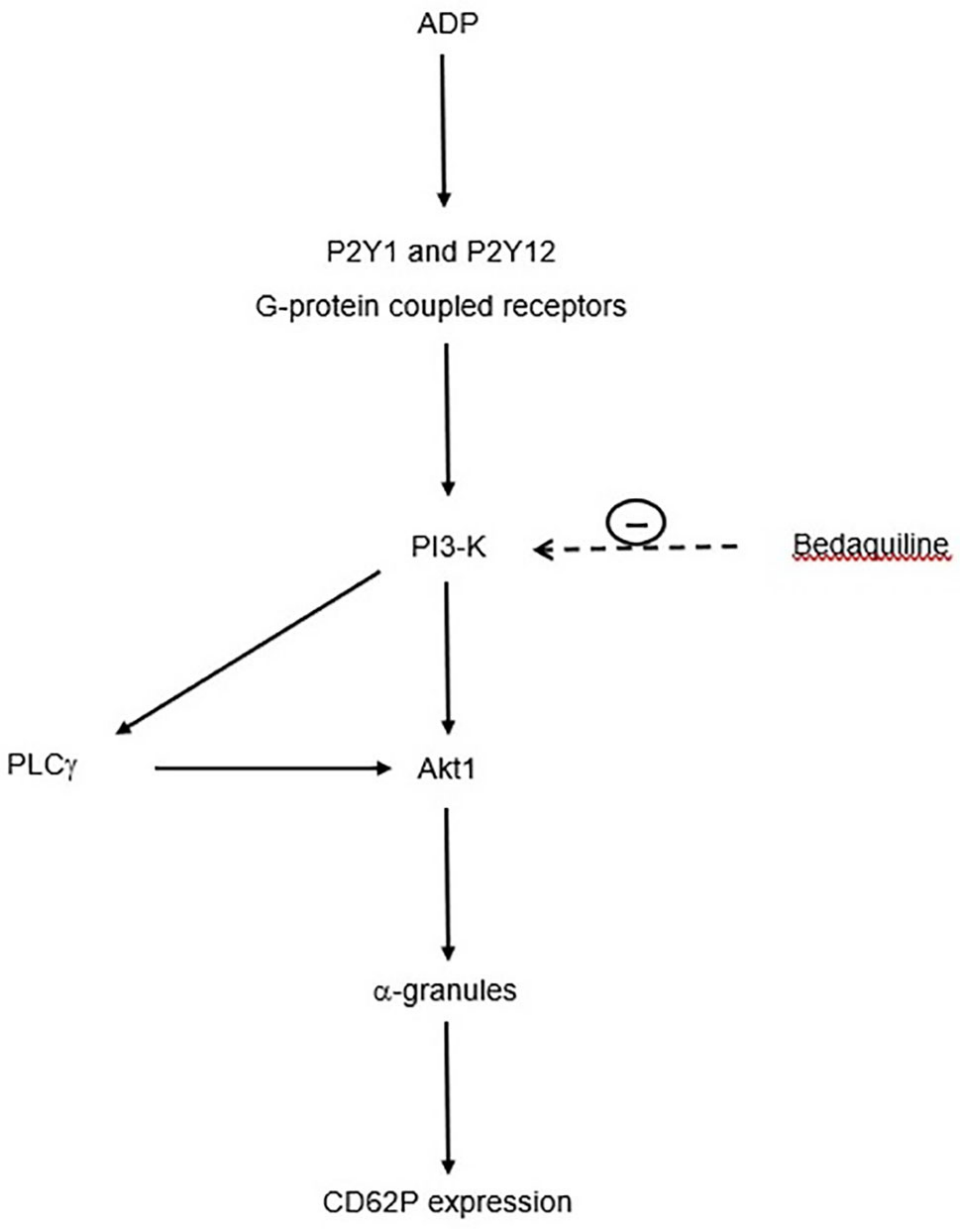
Proposed mechanism by which bedaquiline inhibits upregulated expression of CD62P by ADP-activated platelets. Interaction of ADP with platelet P2Y1 and P2Y12 receptors results in activation of PI3-K (phosphatidylinositol 3-kinase), which turn, activates phospholipase C-γ (PLCγ) and Akt1. These events lead to mobilization of α-granules and upregulation of expression of CD62P. The target of bedaquiline, PI3-K, is indicated by the **<- - - -** symbol.

With respect to the mechanism by which wortmannin inhibits mobilization of intracellular Ca^2+^ following activation of platelets with ADP, it is noteworthy that PI3-K mediates activation of the γ-isoform of PLC (29, 30), which is prominently expressed in human platelets (31). In this context, activation of PLCγ by PI3-K may represent an auxiliary mechanism of Ca^2+^ signaling underpinning the differential effects of bedaquiline, U73122, and wortmannin on intracellular Ca^2+^ fluxes (32). This mechanism is summarized in **Figure 7**.

The apparent link between bedaquiline and inhibition of PI3-K is strengthened by the fact that bedaquiline is a cationic amphiphilic drug with a high affinity for the anionic phospholipid constituents of the plasma membrane (33, 34), which may result in interference with membrane structure and function (35, 36). In this context, cationic amphiphilic drugs have a particularly high affinity for membrane phosphatidylinositol 4,5-biphosphate [PI(4,5)P2], the substrate for PI3-K, possibly interfering with access of this enzyme to its phospholipid substrate (37). Bedaquiline-mediated preferential interference of access to PI3-K to its target 3’-hydroxyl group on the inositol ring of platelet membrane PI(4,5)P2 may therefore underpin the selective inhibitory effects of this agent on ADP-mediated activation, of these cells. Alternatively, or possibly interactively, molecular modeling and docking studies have recently revealed that certain types of cationic amphiphiles also bind to and inactivate the active enzymatic site of PI3-K (38). We do concede, however, that mechanisms other than those proposed here may also be operative in the setting of bedaquiline-mediated suppression of platelet reactivity following interaction of ADP with the platelet purinergic P2Y1 and P2Y12 receptors.

Antagonism of P2Y12 receptors with agents such as clopidogrel and ticagrelor is a well-recognized strategy in the prevention and treatment of myocardial infarction and stroke (39). In this context, it is noteworthy that engagement of the platelet P2Y12 receptor by ADP is linked to activation of PI3-K and phosphorylation of Akt1 (40). If the anti-thrombotic activities of bedaquiline described in the current study, albeit in an *in vitro* setting, are evident in the clinical setting of the extended therapy of MDR/XDR-TB, they may contribute to counteracting the disease-related cardiac dysfunction associated with chronic inflammation and a pro-thrombotic state. This contention may, however, be offset by the aforementioned inhibitory effects of bedaquiline on cardiomyocyte Kv.11.1 channels (4), possibly together with suppression of the activities of various types of cardiac inward rectifier channels that are dependent on channel-PI(4,5)P2 interactions (41), as well as on the anti-arrhythmogenic activity of cardiomyocyte PI3-Kα (42, 43).

## Conclusion

In conclusion, the findings of the current study have identified a novel, potentially beneficial, anti-platelet activity of bedaquiline, which is achieved *via* selective interference with ADP-mediated platelet activation, seemingly involving inhibition of PI3-K. However, the clinical significance of this platelet-targeted, secondary activity of the antimicrobial agent, if any, remains to be established. We concede that testing the effects of bedaquiline in combination with other potentially cardiotoxic MDR/XDR agents *in vitro*, as well as investigating other PI3-K-linked intracellular signaling, mechanisms, together with pre-clinical assessment in murine models of experimental TB, would have added value to our study. Nevertheless, our findings provide a basis for future research of this type.

## Data Availability Statement

The raw data supporting the conclusions of this article will be made available by the authors, without undue reservation.

## Ethics Statement

The studies involving human participants were reviewed and approved by the Research Ethics Committee of the Faculty of Health Sciences, University of Pretoria Approval number: 116/2017. The patients/participants provided their written informed consent to participate in this study.

## Author Contributions

All authors contributed significantly to the conceptualization and planning. GT, AT, MC, JGN, HS, and RA performed the laboratory investigations. GT, AT, and RA contributed to the analysis of the data and preparation of the figures, while all authors contributed to the interpretation of the data and compilation of the manuscript. All authors contributed to the article and approved the submitted version.

## Funding

The study was supported from a grant awarded to MC Cholo by the National Health Laboratory Research Trust of South Africa (Grant: 004 94648), while C. Feldman is supported by the National Research Foundation of South Africa.

## Conflict of Interest

The authors declare that the research was conducted in the absence of any commercial or financial relationships that could be construed as a potential conflict of interest.
